# Impacts of Lubricant Type on the Densification Behavior and Final Powder Compact Properties of Cu–Fe Alloy under Different Compaction Pressures

**DOI:** 10.3390/ma15165750

**Published:** 2022-08-20

**Authors:** Nada S. Korim, Ayman Elsayed, Lianxi Hu

**Affiliations:** 1 School of Material Science and Engineering, Harbin Institute of Technology, Harbin 150001, China; 2 Faculty of Energy Engineering, Aswan University, Aswan 81528, Egypt; 3 Powder Technology Department, Central Metallurgical Research and Development Institute, Cairo 11865, Egypt

**Keywords:** Cu–15Fe alloy, powder metallurgy, solid lubricants, densification behavior, mechanical properties, friction, wear

## Abstract

A Cu–15Fe alloy was fabricated using a powder metallurgy (PM) route, with the addition of different solid lubricants (i.e., paraffin wax (PW) and stearic acid (SA) as well as their composites (PW+SA)). Green compacts were produced via cold compaction at different pressure levels of 50 MPa, 200 MPa, and 350 MPa, then sintered for 60 min under vacuum at 1050 °C. The systematic evolution of the densification, porosity, and pore-size behavior were studied. Vickers Hardness Tests were used to measure hardness. The analysis of the morphological alterations was performed using scanning electron microscopy (SEM) and X-ray diffraction (XRD) techniques. Moreover, under dry sliding conditions, pin-on-disk wear tests were conducted in order to determine tribological properties such as the coefficient of friction (µ), specific wear rate (K), and friction temperature gain. Results revealed that the lubrication process and compaction pressure play a crucial role in defining the characteristics of the final compact. Higher sintered densities and hardnesses were achieved at 50 MPa when PW was used as a solid lubricant, and became reduced as the compaction pressure increased. In contrast, in the case of SA, higher sintered densities and hardnesses were obtained at a compaction pressure of 350 MPa, and increased with increasing pressure. Moreover, PW samples exhibited lower coefficients of friction and wear properties. The addition of SA improves the wear loss of friction materials as well as their coefficients of friction. Compared to blank and PW samples, SA samples show a nearly 50% reduction in wear rate.

## 1. Introduction

Owing to expansion in various fields in our lives over the last 50 years, from communications, medicine, microelectronics, industrial machinery and equipment, etc., global demand for copper alloys (Cu) has tripled as a result of these materials’ ability to adapt to many applications with their high strength with high conductivity [1,2,3]. Cu metal is the best non-precious metal conductor of electricity as well as being the safest, thus it is used widely in commercial and residential building wiring [4]. In addition, Cu alloys are an essential component of energy-efficient generators [5], motors [6,7], transformers [8], and renewable energy production systems [9]. Renewable energy sources such as solar, wind, geothermal, fuel cells, and other technologies, are all heavily reliant on Cu alloys due to their excellent conductivity [10,11]. The copper infrastructure of ordinary telephone wire plays a key role in telecommunications and technologies worldwide; Cu products are used in domestic subscriber lines, wide and local area networks, mobile phones, and personal computers [12,13]. Cu is also extensively used in other electronic equipment, including wires, transformers, connectors, and switches. Cu is used in all major forms of transportation, including boat and ship hulls, automobiles, and trucks [14,15]. Moreover, Cu alloys are well suited for manufacturing products such as gears, bearings, and turbine blades [16,17,18]. Due to Cu’s superior heat transferability and resistance to extreme environments, it is ideal for heat exchange equipment, pressure vessels, and vats. However, due to their low hardness and poor abrasion resistance, their application in high-strength environments is severely limited [19]. One of the most common methods to improve the mechanical properties of Cu is to add different trace elements to it, such as iron (Fe) [20], titanium (Ti) [21], nickel (Ni) [22], silicon (Si) [23], and chromium (Cr) [24]. Cu–Fe alloys are used in a variety of electrical devices, such as semiconductor lead frames, electrical connectors, and electrical fuses. The reason for this is because such alloys have a low thermal expansion coefficient and are highly durable and strong [25]. A number of studies have been conducted on Cu–Fe alloys at different solidification rates and microstructure evolutions, such as liquid separation [26], mechanical alloying [27], spray deposition [28], double-melt mixed casting [29], and powder metallurgy (PM) [30], for the purpose of fabricating ideal alloys.

PM techniques can be applied in several material fields as well as industries, such as aeronautics, astronautics, marine industries, and automobiles [31]. As well, many engineering products are manufactured through this method, such as gearboxes and transmissions for automobiles, raw carbides for motors, magnets and soft magnetic materials, and ceramics [32,33]. Generally, the major stages of PM production processes usually involve powder mixing as the first step, followed by powder forming (or consolidation of metal or alloy powders by applying uniaxial or biaxial pressure) [34], and finally, sintering processes [35]. One of the key stages of PM production is powder compaction, which plays a critical role in determining the final performance of parts. Loose powders are flattened and compressed through a compaction process that uses tools such as compaction punches and dies [36]. Final PM-product properties, such as hardness, rigidity, and dimensional stability, all depend upon multiple factors, including powder type and size, amount and type of lubricant, pressing pressure, sintering temperature, and time, as well as finishing techniques [37].

During powder compaction, tools are used to apply force and pressure to cause deformation of the powder, as well as relative motion between the tool and the specimen. An important consideration, therefore, is the friction that exists at the tool/specimen interface, between the die walls and specimen, and among powder particles. Production rates, tool design, die dimensions and process optimization all depend on the ability to determine and control friction between the tool and compacted powder, and between the die wall and compacted powder. Furthermore, the specific friction conditions depend on several factors, which include contact area, contact pressure, surface finish, speed, type and amount of lubricant, and temperature [38]. From a mechanistic perspective, the friction between the sample and the die walls and/or the tool can be attributed to (1) abrasion, the force necessary to plow the peaks of a harder material through a softer one, and/or (2) adhesion, the force required to rupture microscopic weldments formed between the two materials. A second phenomenon that is directly related to friction is surface deterioration or wear [39]. In general, since the compacted powder only interacts with the tool during its own operation, any wear experienced by the final product in its forming operation is usually not objectionable. It is inevitable that the tools will wear out; however, at this point, the final product dimensions will be affected. Additionally, increased frictional resistance (which increases power requirements and reduces process efficiency), a poor finish on the product as well as a loss of production during tool changes are also consequences of tool wear [38].

Lubrication is often the key to success in powder compaction operations [40,41,42,43]. Lubricants are generally chosen as important additives in the powder metallurgy industry, in which their ability to decrease the friction among powder particles as well as between the powder particles and the die wall can prevent the tool wear. Secondary considerations may include the ability of lubricants to act as a thermal barrier (keeping the heat in the workpiece and away from the tooling) [44], the ability to act as a coolant (removing heat from the tools) [45,46], and the ability to avoid corrosion and rust on the formed product [47]. As a result, the powder is more effectively pressed, greater density is achieved, and the mechanical properties of products are enhanced [48,49,50]. Moreover, adding lubricant also reduces the ejection force of a mold, allowing parts to be more easily ejected from it, and hence prolonging equipment life [51,52,53,54,55,56,57].

A significant amount of research has been done on lubricants and lubrication processes for PM materials. Internal lubrication differs greatly from die wall lubrication in terms of its lubrication mechanisms. Therefore, analyzing the research objectives and methodologies separately is necessary. Regarding the internal lubrication mechanism on the powder compaction process, it is essential to identify the most appropriate internal lubricant composition and additive content that is easy to remove. M.M. Rahman [58] examined the mechanical properties and microstructures of components manufactured above ambient temperatures as a function of the lubricant content. The results revealed that the characteristics of the final product were influenced by lubricant content and mixing time. Maja Kokunešoski [59] used methyl methacrylate (MMA) as an acrylic binder in the preparation of ceramic powder of alumina for pressing to make alumina flakes more machinable. The results revealed that the machining of green bodies, prepared with MMA, can be used to make complex ceramic products, such as screws with nuts, and has great potential for commercialization. Secondly, for die wall lubrication, it is important to determine by how much a lubricant coating reduces the friction coefficient of the die wall. Ejection behavior is an excellent indicator of the advantages of applying a lubricant. For instance, C. Machio [60] studied the effect of three commonly used die wall lubricants on the cold compaction behavior of titanium hydride powder. It appeared that cold compaction is immediately improved by lubrication when compared to the dry process. M. Larsson [61] presented the synergies between the lubricant content, the lubricant efficiency, and the tool die temperature in order to show that compaction with a warm die is an effective process to make highly dense PM components. Daniel Toledo dos Santos [62] studied the effect of the die wall lubrication on the green strength and the tensile properties of high-temperature sintered and sinter-hardened low alloy steel. As a result, sintered and sinter-hardened materials displayed superior tensile properties for die wall lubrication compared to those treated with bulk lubrication.

Comparative studies of lubricants have been made in various publications. A comparison of the most common lubricants used in the PM industry including Acrawax, zinc stearate, and Kenolube (a mixture of the prior two components) was conducted by Y. C. Lin [63]. Based on the results, Kenolube offers the lowest ejection force, while Acrawax is most effective at strengthening green compacts, and zinc stearate yields the highest green density. Additionally, the choice of lubricant and its concentration depends upon the green compact requirements. Furthermore, the amide lubricants such as erucylamide have low melting points which qualify them to be excellent lubricants [64]. However, it may cause agglomeration and reduce the powder flow rate, and could even result in powder not flowing [65]. The efficacy of zinc stearate and paraffin wax were evaluated by Y. Taniguchi [66] as an internal lubricant or die wall lubricant for improving the density uniformity of iron and pre-alloyed stainless steel powder compaction. Based on the results, paraffin wax was significantly better at lubricating die walls than zinc stearate. In order to achieve a more uniform green density, the performances of stearic acid and magnesium stearate as lubricants added to titanium powder compacts were compared by J. Lou [67]. Additionally, measurements of the mechanical properties were made by cutting selected samples from each sintered compact for the tensile test. According to the results, variations in mechanical properties are a function of pore size, aspect ratio, and preferred orientation. Besides, the addition of stearic acid as a lubricant is the best choice due to its ability to distribute the pores evenly, to reduce pore size, and provide an acceptable level of oxygen pick-up compared with magnesium stearate.

Based on the above literature, it is evident that the compaction pressure level greatly affects the densification behavior and final compact properties. Furthermore, the lubrication process is essential to the powder compaction success. Thus, further research is required to understand how lubricant types, along with compaction pressure levels, affect PM products. Therefore, the main objectives of this research are the following:

- Study the effects of different solid lubricants, PW and SA and their composites, along with different compaction pressure levels on the densification behavior and final compact properties of Cu–15Fe alloys based on the PM route.
- Evaluate and analyze the tribological properties of final compacts under dry sliding conditions, such as coefficients of friction, specific wear rates, and friction temperature gains.
- Investigate the porosity behavior, mechanical properties, microhardness, and microstructural changes in the sintered samples.

## 2. Experimental Procedure

### 2.1. Materials and Powder Compositions

In this study, electrolytic copper powder of 99.8% purity and 0.8-micrometer particle size, and iron powder of 99.9% purity and particle sizes of 250 µm (both provided by Oxford Lab Fine Chem LLP), were used as raw materials. Additionally, commercial lubricants used in this study were stearic acid and paraffin wax. The copper powder was manually blended with 15 wt.% iron powders for about half an hour, followed by mechanical alloying for 2 h in a planetary ball mill (PQ-N2) with a speed of 300 rpm. In addition, argon gas was pumped into the milling jar before operation in order to prevent powder oxidation. With a ball to powder weight ratio of 3:1, hardened steel balls were used as grinding media. Then, lubricant was added to the powder mixture. Table 1 displays the amount of composite lubricant in the Cu–15Fe powder, as well as the mixture ratio of the composite lubricant.

A drying oven was then used to mix the Cu–15Fe composite powder with paraffin wax for approximately 1 h at temperature 90 °C while continually hand stirring. On the other hand, stearic acid was dissolved in ethanol and then mixed with Cu–15Fe composite powder; the resulting mixture was wetly blended till solvent evaporation was complete. Then, the mixture was dried in a drying oven at 70 °C with constant hand stirring for 1 h.

**Figure 1:**
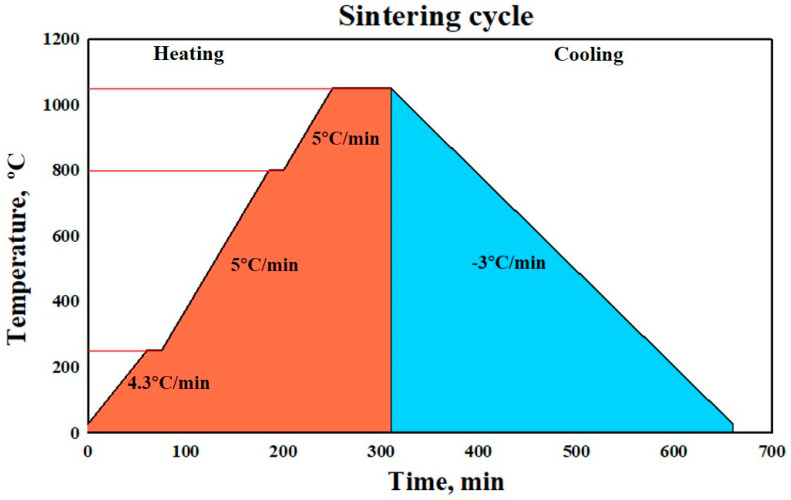
Sintering cycle used for all samples.

Green samples were prepared by cold pressing in a cylindrical hardened steel die (Ø 10 mm × 4 mm length) under various values of pressure (50 MPa, 200 MPa, 350 MPa). The samples were kept at the highest pressure for one min at room temperature. The green compacts were then sintered at 1050 °C for one hour in a vacuum atmosphere. Sintering was carried out under a continuous primary vacuum atmosphere in a vacuum sintering furnace that located in Central Metallurgical Research and Development Institute in Egypt (model furnace of 121212-50, GEA/Vacuum Industry, USA). The sintering cycle applied for all experiments is shown in Figure 1, where it includes the following steps:

- Heat up to 250 °C for 1 h and hold for 15 min for wax removal.
- Heat up to 600 °C at 5 °C/min and hold at 600 °C for 15 min for internal stress relief.
- Heat up to sintering temperature of 1050 °C at 5 °C/min and hold for the sintering time of 60 min.
- Slow cool at 3 °C/min down to room temperature in order to prevent cracking.

### 2.2. Microscopic, Porosity and Microhardness Studies

A standard metallography procedure was used to prepare the surfaces of the sintered samples. The specimens were coarsely polished with abrasive papers of 600, 800, 1000, 1200, 1500, and 2000 grit. Fine polishing was carried out using a 0.3-micrometer alumina paste. After polishing, the samples were etched in an aqueous solution containing 25 g of ferric chloride (${\mathrm{FeCl}}_{3}$), 10 mL of hydrochloric acid (HCl), 100 mL of ethyl alcohol ($C_{2}H_{5}\mathrm{OH}$), and 100 mL of $H_{2}O$. The etching was performed for 30 s, and afterwards the samples were rinsed in water and blow-dried. The samples’ metallography before and after etching were performed using an optical microscope (Nikon, Tokyo, Japan).

The optical micrographs were used to demonstrate the performance of Cu–15Fe sintered samples as a function of multiple compaction pressures levels and various lubricants. ImageJ software was used to measure the porosity and determine the pore size and distribution from the optical pictures. A scanning electron microscope (SEM) was used to observe the microstructures. A Vickers hardness tester was also used to evaluate the microhardness variations of the alloy under the influence of different types of lubricants and various pressure levels by applying a one newton load. The measurements were taken as an average of five test results for five different locations on each specimen.

### 2.3. Tribological Evaluation

Tribological properties were characterized using a pin-on-disk tribometer (model: SAE-J661) under dry sliding conditions at an ambient temperature of 30 °C. The pin sample surface was prepared by polishing with 1200-grit abrasive paper and cleaning with acetone. The pin installed in the specimen holder was vertically positioned against the disk which was made from alloyed grey cast iron. Prior to testing, the contact surface of the disk was polished by hand with 1000-grit abrasive papers, cleaned with acetone to remove the surface contaminants, and dried. Under the friction surface of samples, a thermocouple was fixed at a distance of 2 mm to measure the temperature near the friction surface.

#### 2.3.1. Coefficient of Friction (µ)

The coefficient of friction between samples and cast iron disc was continuously recorded through a frictional force transducer attached to a sliding wear testing machine. The µ data were generated at the dry friction conditions for a sliding distance of 7500 m at 4.32 m/s relative disc speed, with a normal load of 4 kg. The µ is acquired by dividing the measured tangential force $F_{t}$ by the applied normal load $\;F_{n}$.

(1) µ=FtFn,

#### 2.3.2. Specific Wear Rate

The specific wear rate  K (also known as a dimensional wear rate) for the samples is simply calculated by dividing the volume loss $V_{l}$ by the product of the normal applied load $\;F_{n}$ and the sliding distance  d.

(2) K=VldFn,

A precision balance with a sensitivity of 10^−4^ g was used to weigh the samples before and after each test to calculate the wear degree undergone by the material (mass loss). The volume loss $V_{l}$ was the mass loss ∆m divided by the bulk density of specimen  ρ.

(3) Vl=∆mρ,

This test was carried out for 29 min for all samples, commensurate with a sliding distance of 7500 m.

## 3. Results and Discussion

### 3.1. The Green and Sintered Densities of Compacts

**Figure 2:**
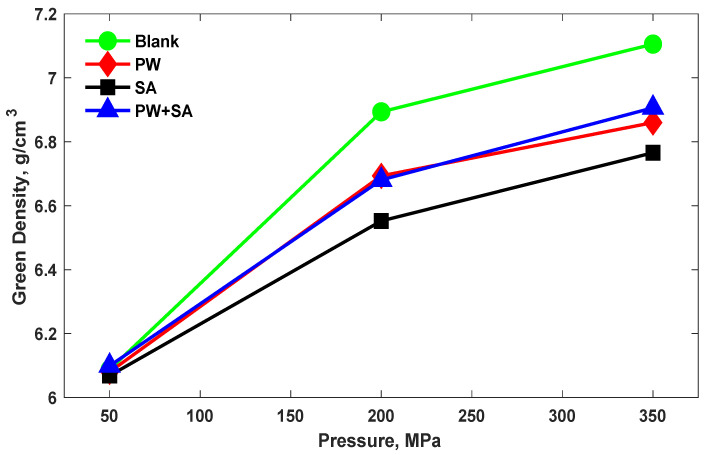
The effects of various compaction pressure levels of the green density of Cu–15Fe green samples with and without different lubrication types.

The green density of Cu–15Fe samples under different lubricants along with the different pressure levels used are shown in Figure 2. It can be seen that the green density of the compacted samples increases with increasing the compaction pressure for all samples. With an increase in pressure from 50 MPa to 200 MPa, the green density of blank samples improved by 13.2%, but this rate dropped to 3% with an increase in pressure to 350 MPa. Additionally, the green density of PW samples rose with increasing the pressure from 50 MPa to 200 MPa by 10%, and by 2.5% from 200 MPa to 350 MPa. Furthermore, the increase in green density for SA samples was 8% higher when the pressure increased from 50 MPa to 200 MPa, and it was 3.2% higher when the pressure increased to 350 MPa. Similarly, with a rise in the pressure from 50 MPa to 200 MPa, green density in PW+SA samples increased by 9.5%; at 350 MPa, it rose by 3.4%. It is evident that the rate of increasing the green density from 50 MPa to 200 MPa is greater than the rate of increasing the green density from 200 MPa to 350 MPa.

According to the results recorded in Figure 2, increasing compaction pressure increases the plastic deformation of the green compact which was expected to improve the density of Cu–15Fe sintered samples. It is worth mentioning that all the samples that contain lubricants showed values of green densities that are lower than those of blank samples. The reason for this is because the densities of lubricants are much lower than those of the alloys.

**Figure 3:**
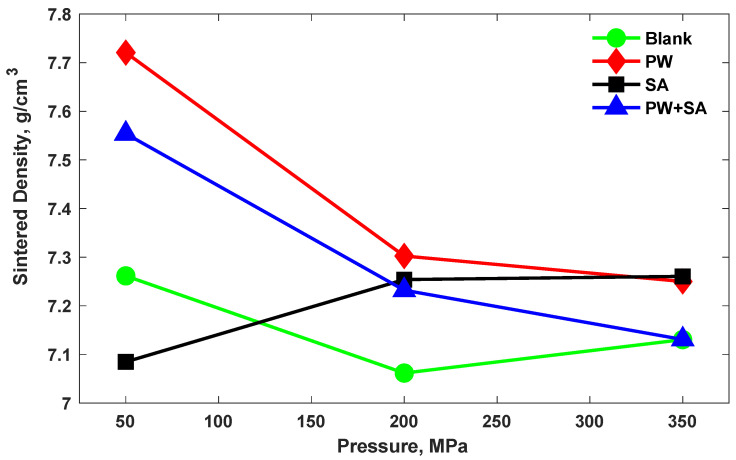
The effects of various compaction pressure levels on the sintered density of Cu–15Fe sintered samples, with and without different lubrication types.

On the other hand, the density of Cu–15Fe sintered samples under several pressure values is presented in Figure 3. It can be seen that the densification of both the PW samples and PW+SA samples significantly decreased with increasing compaction pressure. The sintered density of PW samples decreased by 5% when the pressure was raised from 50 MPa to 200 MPa, and by 0.7% with increasing pressure from 200 MPa to 350 MPa. Likewise, the sintered density decreased by 4% and 1.4% in PW+SA samples with increasing pressure. The reason for this is because the lubricant does not easily exit out of the material during sintering when the pressure is high, since the pores are not interconnected. On the contrary, the sintered density of the SA sample improves by 2.4% and 0.1% as the compaction pressure is increased, which is caused by the lower melting point of SA compared with that of PW; this increases its ability to escape from narrower pores. It also appears that the PW sample has the highest sintered density at a pressure of 50 MPa (the lowest pressure) by 5.7% in comparison with the blank sample. Moreover, the SA sample has the largest sintered density under a pressure of 350 MPa (the highest pressure) by 2% in comparison with the blank sample. At a pressure of 50 MPa, the sintered density of the PW+SA sample is higher than that of the SA sample and lower than that of the PW sample. On the other hand, the sintered density of the PW+SA sample when subjected to a pressure of 350 MPa is the lowest of all the samples tested, and it is the same as the sintered density of the blank sample.

According to analysis of the densification behavior after the sintering process, it can be noticed that using PW as a lubricant improves the sintered density particularly when lower pressures are used compared to higher pressures. Considering the PW constituents, it has non-polar bonds and separate chains that are held by relatively weak van der Waals forces. Through the sintering process, the PW bonds are weakened and their lengths are increased; then, the wax evaporates and flows past the sample particles and the furnace insulation to exit the hot zone. In the low pressurized samples, a large amount of PW can escape, causing a reduction in volume and a rise in density. In contrast, in highly pressurized samples, a large amount of wax cannot leave the sample, and consequently microcracking occurs as the wax-laden vapors come in contact with the elements, forming carbon-rich domains that increase the volume of the specimen, hence decreasing its density. According to these results, PW can be considered a suitable lubricant for Cu–15Fe alloy with an acceptable green density and an extraordinary sintering density at low pressures. However, at high pressures, SA is a suitable lubricant for the Cu–15Fe alloy.

### 3.2. Porosity of Sintered Compacts

**Figure 4:**
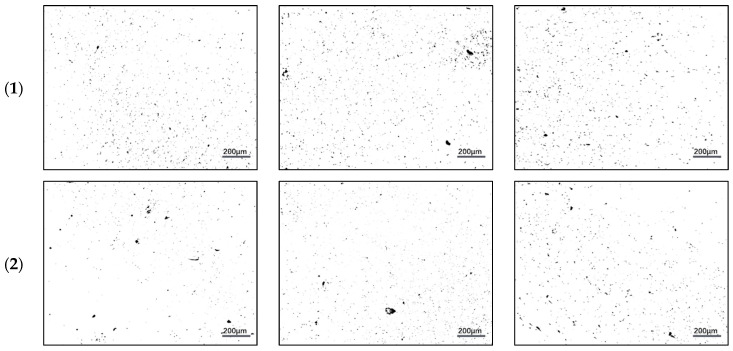

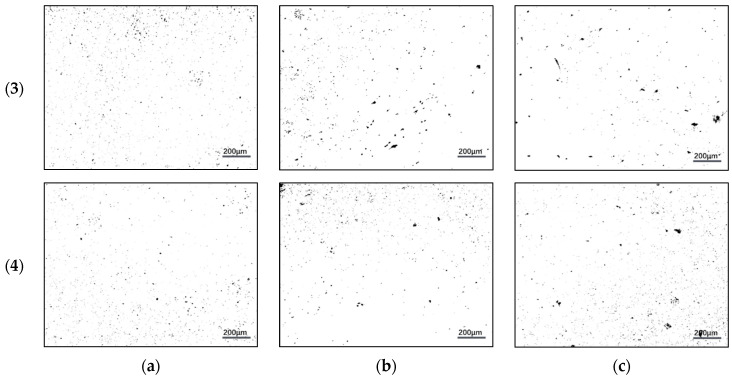
Optical micrographs of sintered Cu–15Fe powder compacts: (**1**) blank sample, (**2**) PW sample, (**3**) SA sample, and (**4**) PW+SA sample under different pressures: (**a**) 50 MPa, (**b**) 200 MPa, and (**c**) 350 MPa.

ImageJ software was used to conduct an investigation of how the area porosity changes and the distribution of pores varies in the sintered Cu–15Fe powder compacts subjected to a variety of lubricant types while under various compaction pressures. Figure 4 depicts the sintered samples’ pore structure and distribution, while Table 2 lists the changes in the area porosity that may be detected as a result of the sintering process. When examining the blank sample, it is possible to observe that the average size of pores (from Figure 4(a1–c1)) as well as the percentage of area pores (from Table 2) became large as the pressure rose from 50 MPa to 200 MPa; however, when increasing the pressure to 350 MPa, the rate of percentage of area pores became slightly lower than it had been at a pressure of 200 MPa (from 1.24% to 1.1%). In addition, the percentage of area pores at 50 MPa is the lowest in the PW sample (0.5%), but the average size of pores in this sample at 50 MPa is greater than that in the blank sample, as shown in Figure 4(a2–c2). As the pressure is increased (in 200 MPa and 350 MPa), it is possible to observe an increase in the percentage of area pores (0.77% and 0.85% respectively) while simultaneously observing a drop in the average size of pores.

Furthermore, as indicated in Table 2, the rate of area porosity in the SA sample reduced whenever there was an increase in pressure. Also noteworthy is that the pressure caused an increase in the average pore size in the SA sample, as can be seen in Figure 4(a3–c3). In addition, it is possible to see that an increase in pressure from 50 MPa to 350 MPa causes an increase in both the average size of the pores as well as the rate of area porosity in the PW+SA sample (Figure 4(a4–c4)).

### 3.3. Hardness

**Figure 5:**
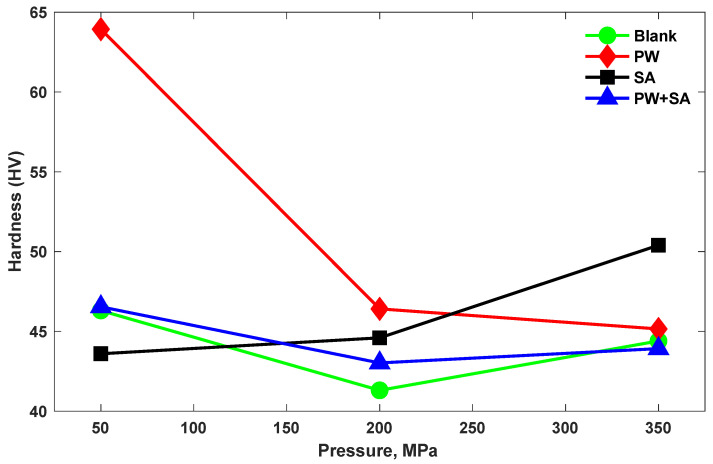
Hardness of the Cu–15Fe sintered composite powder samples, without and with different lubrication types, at various compaction pressures.

Vickers hardness tests are employed to determine the bulk hardness of sintered samples. Figure 5 shows the variation of hardness for all the sintered samples at the various compaction pressure levels. The plot of PW samples shows that with raising of the compaction pressure, the measured hardness decreases by 27.4% and 2.7%, from 50 MPa to 200 MPa and 200 MPa to 350 MPa, respectively. Additionally, according to a plot of PW samples, the measured hardness reduces by 7.6% as the pressure increases from 50 MPa to 200 MPa and rises slightly by 2% as the pressure is further increased to 350 MPa. Moreover, for the blank samples, with increasing pressure from 50 MPa to 200 MPa, the measured hardness decreases by 10.8%, and with increasing pressure from 200 MPa to 350 MPa, the hardness increased by 7.5%. Additionally, the hardness values of the SA sample increase as the compaction pressure increases by 2.3% and 13% from 50 MPa to 200 MPa and 200 MPa to 350 MPa, respectively, which agrees well with the findings for density versus compaction pressure of SA lubricant.

The above-mentioned decrease in hardness at higher compaction pressures for the PW and PW+SA samples can be explained by the entrapped vapor effect inside closed pores as it formed a carbon-rich environment in the late stages of compaction while trying to escape and vent towards the surface. This leads to a decrease in hardness as a surface property more than for other properties. According to these results, SA was found to be a suitable lubricant material for the Cu–15Fe sintered samples with high hardness values that increased by 13.5% in comparison with the blank sample at higher compaction pressures, while PW was found to be a suitable lubricant material for the Cu–15Fe sintered samples with exceptional hardness values that increased by 38% in comparison with the blank sample at lower compaction pressures.

### 3.4. Microstructure of Sintered Samples

**Figure 6:**
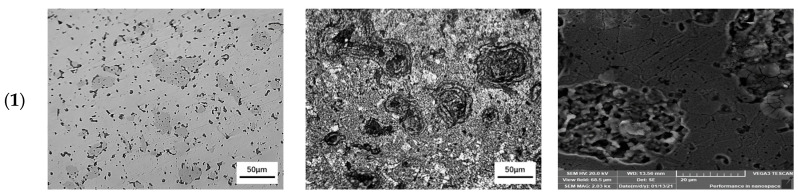

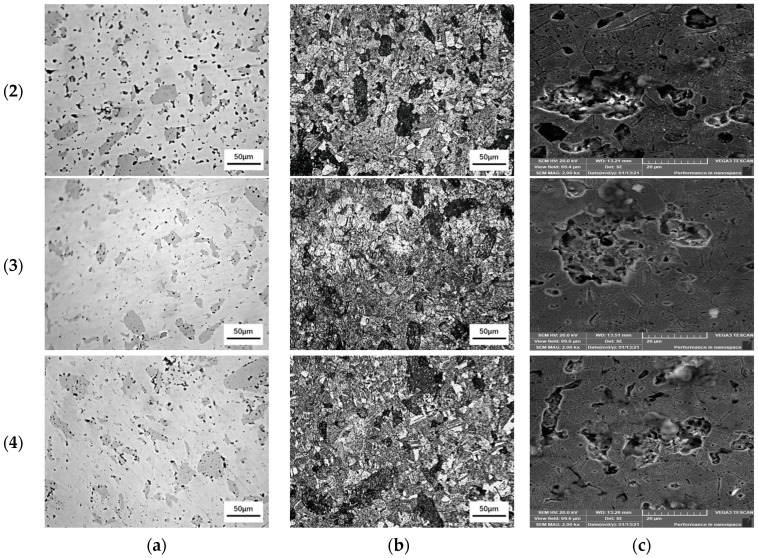
Optical micrographs (**a**) without and (**b**) with chemical etched, and (**c**) SEM micrographs of Cu–15Fe sintered samples at 200 MPa: (**1**) blank, (**2**) PW, (**3**) SA, and (**4**) PW+SA.

Optical micrographs before and after chemical etching, as well as SEM micrographs of the Cu–15Fe sintered samples with different types of lubricant at a compaction pressure of 200 MPa (the middle pressure value was selected as a representative sample), are shown in Figure 6. The microstructure of the blank sample is shown in Figure 6(a1–c1) where the light grey matrix is Cu, the darker gray areas are Fe, and the small dark spots are Cu–15Fe oxides, with a small amount of carbon (resulting from the sintering furnace atmosphere). It can be seen that a uniformly homogenous distribution of Fe particles in the Cu matrix is evident due to proper selection of mixing conditions. A good interface between copper and iron particles is formed, which provides a strengthening effect to the copper matrix. Figure 6(a2–c2) shows the microstructure of the PW sample where it can be noticed that the dark spots tend to be more obvious and bulkier. They can be seen in both the optical and SEM micrographs of this sample in Figure 6(c2). These spots indicate the carbon-rich environment that results from the evaporation of PW during the sintering process.

Figure 6(a3–c3) shows the microstructure of the SA sample where the decrease in the number of pores along with better homogeneous distribution of Fe particles in the Cu matrix result from the presence of SA. The least number and the smallest size of pores can be shown in Figure 6(a3,c3). The microstructure of the PW+SA sample is shown in Figure 6(a4–c4) where the dark spots that appeared in Figure 6(a4) tend to be similar to those shown in Figure 6(a2), but in a lower concentration. These spots result from the amount of carbon released by paraffin wax that was evaporating during the sintering process. Moreover, the percentage of paraffin wax in the composite lubricant was 1%, which is half the percentage used in Figure 6(a2–c2). Thus, the distribution of dark spots in Cu–15Fe composite powder in Figure 6(a4–c4) is more homogeneous than in the powders shown in Figure 6(a2–c2). It can also be seen that these areas in Figure 6(a4) are smaller than the ones found in Figure 6(a2).

**Figure 7:**
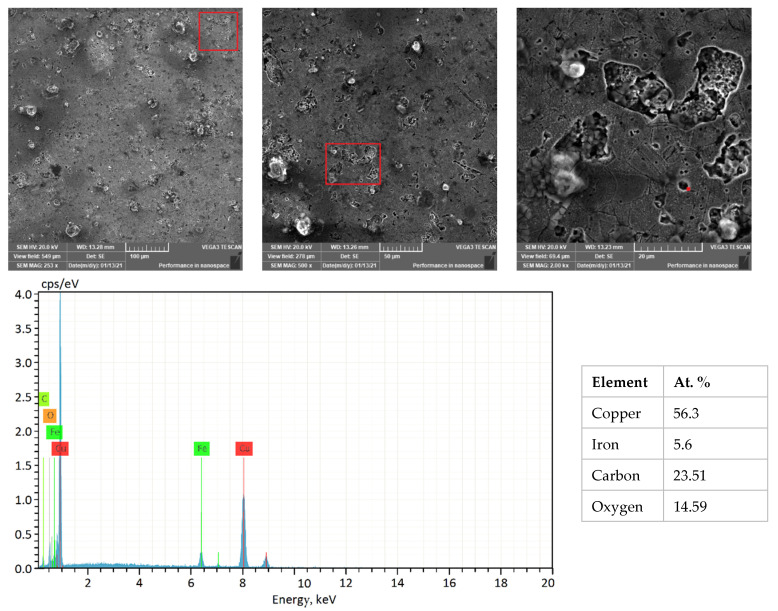
SEM micrographs and the corresponding EDS analysis of the PW+SA sample at 200 MPa.

The SEM micrographs and corresponding EDS analysis of the PW+SA sample at a pressure of 200 MPa are shown in Figure 7. The results of the EDS analysis show that the dark grey areas represent the iron-rich phase that is distributed throughout the Cu-rich matrix. During the sintering process, the paraffin wax evaporates, which results in the diffusion of carbon onto the surface. Additionally, the chemical solution etching process results in the diffusion of oxygen onto the surface. It is important to note that the carbon percentage in the EDS analysis indicates that the lubricant (both PW and SA) is distributed evenly throughout the Cu–15Fe matrix.

**Figure 8:**
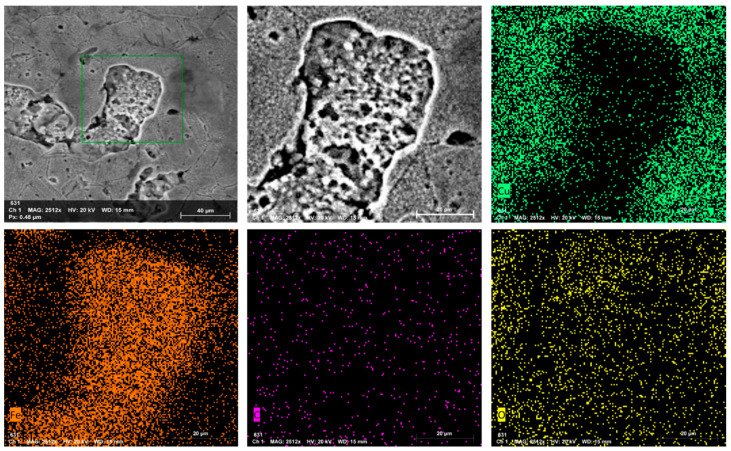
EDS distribution maps of the PW+SA sample at 200 MPa.

The enlarged morphology and corresponding elemental distribution maps of the PW+SA sample at 200 MPa are exhibited in Figure 8. It is important to highlight the presence of the elements of Fe, Cu, C, and O on the surface of the sample that can be observed from the elemental distribution maps. The carbon distribution map shows a uniform distribution on the surface of the composite powder that is similar to the previous EDS results.

**Figure 9:**
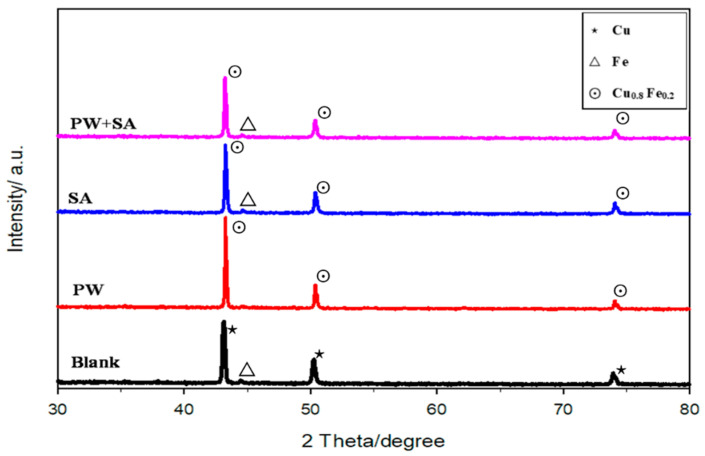
XRD analysis of the Cu–15Fe sintered samples with different amounts of composite lubricant at 200 MPa.

XRD analysis results of the Cu–15Fe sintered samples with different amounts of composite lubricant at a compaction pressure of 200 MPa are shown in Figure 9. The most noticeable pattern is that the recorded peaks correspond to the free FCC copper and BCC iron in the blank sample, while the recorded peaks in the PW, SA, and composite lubricant samples were Cu_0.8_Fe_0.2_ and BCC iron. The new phase of Cu_0.8_Fe_0.2_ demonstrates that iron gradually reacted with copper to form a supersaturated solid solution with the help of the lubricant. The peaks that are specific to the BCC iron phase are less intense, and the Cu and Cu_0.8_Fe_0.2_ diffraction peaks are asymmetric. The peaks that are specific to the BCC iron phase are not found in the PW sample, demonstrating that the paraffin wax lubricant has a good reaction with the Cu–15Fe composite powder.

### 3.5. Tribological Properties

#### 3.5.1. Friction Behavior

**Figure 10:**
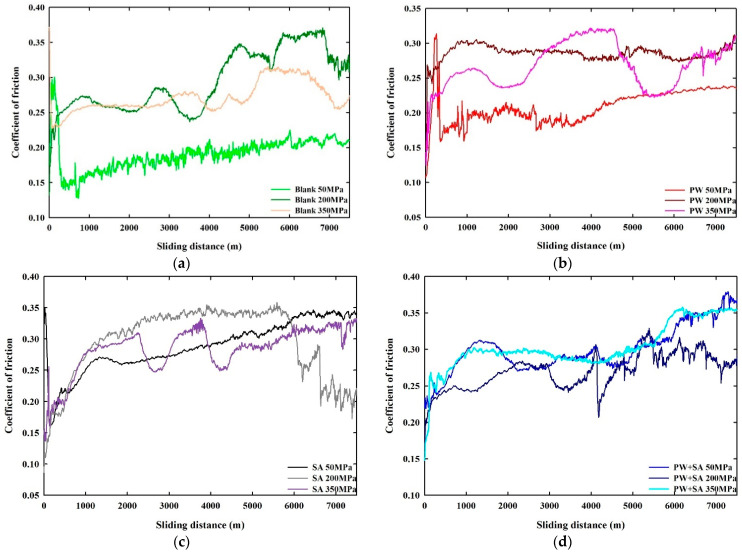
Effect of compaction pressures on coefficients of friction µ as a function of sliding distance for sintered Cu–15Fe samples: (**a**) blank, (**b**) PW, (**c**) SA, and (**d**) PW+SA.

As shown in Figure 10, compaction pressures have a great effect on µ as a function of sliding distance when comparing sintered Cu–15Fe samples under different types of lubricants. The partial contact between the sample and the sliding disc caused µ to be extremely unsettled, initially, for almost all samples. Nevertheless, µ became stable as the sliding distance increased and then proceeded to a steady-state after a certain distance when the sample was in full contact with the disc. In most samples, steady-state conditions were achieved within a sliding distance of 200 m, except for a few samples where steady-state conditions were achieved at a longer sliding distance.

The coefficients of friction data for blank samples under three pressures (50–200–350 MPa) are presented in Figure 10a. When the blank sample reached steady-state, the µ was extremely low, with a range of between 0.13 and 0.23 under 50 MPa of pressure. However, when the pressure was increased to 200 MPa, the µ rose significantly and varied from 0.22 to 0.37. For the blank sample at a pressure of 350 MPa, the µ ranged from 0.22 to 0.32, which was lower than that at 200 MPa pressure. As seen in Figure 10b, µ data of PW samples are displayed at three pressure levels (50–200–350 MPa). It is apparent that once the steady-state condition had been reached, the µ data of the PW samples under different pressure levels increased with increasing pressure. According to the PW sample, µ values ranged from 0.16 to 0.24 under a pressure of 50 MPa. When the pressure was increased to 200 MPa, the µ values rose from 0.23 to 0.31. At 350 MPa of pressure, µ again jumped to 0.22–0.32. As can be seen from these results, the µ values of PW samples were greatly affected by the application of high pressure because of the formation of a layer of carbon on their surfaces.

In Figure 10c, the SA samples at a pressure of 50 MPa showed a µ varying in the range of 0.21–0.35 after achieving stability. As the pressure was increased (200 MPa), the µ range increased to 0.18–0.36. In fact, µ dropped 0.2–0.33 when the pressure was raised to 350 MPa. This is significantly lower than the values for 50 MPa of pressure. A µ graph displaying the results of Cu–15Fe samples that were lubricated with the composite lubricant (PW+SA) is shown in Figure 10d. The µs for PW+SA samples were the highest at a pressure of 50 MPa, and their range was between 0.22 and 0.38. However, increasing the pressure to 200 MPa resulted in a decrease in µ of 0.16–0.33. As the pressure increased to 350 MPa, the µ range increased to 0.15–0.36, which was not higher than that for 50 MPa.

As previously observed, the µ range for the PW+SA sample at 50 MPA was the greatest, while for the sintered Cu–15Fe blank, it was the smallest. As evidenced by the PW samples, the rate of µ increased as the pressure increased from 50 MPA to 350 MPa. The variation in µ for the SA sample at a pressure of 350 MPa was greater compared to the PW sample at the same pressure. 

#### 3.5.2. Wear Characteristics of Sintered Samples

**Figure 11:**
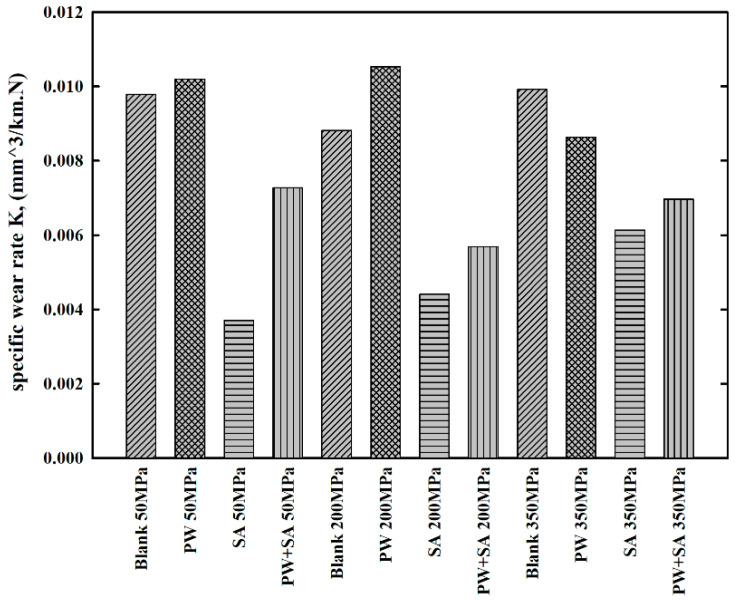
Variations in specific wear rates for sintered Cu–15Fe-based composite samples under different compaction pressure levels.

Under different compaction pressure levels, Cu–15Fe sintered samples were subjected to regular measurements of specific wear rate (K) at regular intervals as a function of sliding distance up to 7500 m when lubricated with various types of lubricant. The average K for each of the samples was measured for the entire test run, and the results are offered in Figure 11. A maximum average K of 0.011 ${\mathrm{mm}}^{3}/\mathrm{km}.N$ was recorded, which is considered to be extremely low. Low wear rate results from the formation of a thin, strong, smooth, and solid oxide friction film. However, it caused a high and unpredictable coefficient of friction. Oxide friction films are formed by the interaction of oxygen and water with liquids and solids on the surface during the processes of plastic deformation and oxidation. When the disc material was subjected to frictional load and heat, copper and iron tend to fuse with it, facilitating the formation of the oxide film as well as heat generation. A stable film of compact oxide is formed during sliding between the sample and disc as the material is transferred back and forth between them. As a result of the film covering the friction surface, the wear rate decreases and the friction surface becomes smooth or less coarse. Therefore, the average Ks for blank samples are 0.01, 0.009, and 0.01 mm^3^/km.N under three different pressures of 50, 200, and 350 MPa, respectively.

It can be seen that using paraffin wax as a lubricant increases the average Ks of PW samples (0.0102 and 0.0105 mm^3^/km.N) at low pressures (50 and 200 MPa, respectively). However, in raising the pressure to 350 MPa, the average K of the PW samples (0.0086 mm^3^/km.N) drops to slightly below that of the blank sample at the same pressure. The addition of paraffin wax into the base material stabilized and decreased µ by the formation of a glazed lubrication film. Higher porosity in the sintered PW sample, due to the existence of carbon, caused a large volume loss under sliding action, and hence the extremely high wear rate was observed. SA samples showed substantial reductions in specific wear rates, with average Ks of 0.0037, 0.0044, and 0.006 mm^3^/km.N, respectively. Compared with blank and PW samples, Cu–15Fe samples that were lubricated with SA show a nearly 50% decrease in wear rate. For Cu–15Fe samples lubricated with composite lubricants, average K decreases with PW and increases with SA, which is due to the combined action of PW (the carbon from the evaporation of paraffin wax) and SA. Average K values of PW+SA samples are 0.0072, 0.0057, 0.007 mm^3^/km.N, respectively. Meanwhile, the SA samples and the PW+SA samples exhibited the highest µ values under the influence of various pressure levels, due to hard and strong stearic acid particles.

#### 3.5.3. Friction Temperature Gain

**Figure 12:**
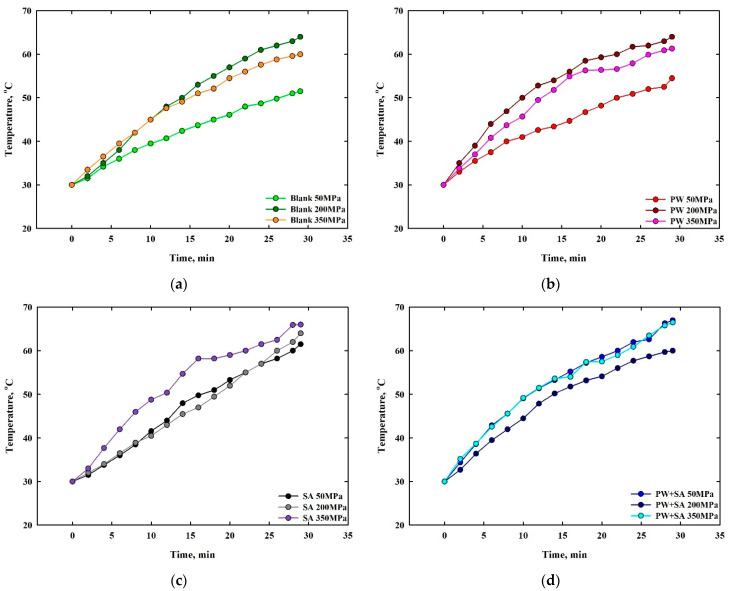
Friction temperature gains as a function of sliding distance for sintered Cu–15Fe-based composite samples: (**a**) blank, (**b**) PW, (**c**) SA, and (**d**) PW+SA, under different compaction pressure levels.

A comparison of friction temperature gains for sintered Cu–15Fe composite samples with a given sliding distance, under different lubricants, is illustrated in Figure 12. The friction temperature gain that was measured for the blank sample is displayed in Figure 12a. The friction temperature gain of blank samples increases with progressively increasing pressure from 50 to 200 MPa, then barely begins to dip at a pressure of 350 MPa. According to the analysis of the previous results, the variance of the friction temperature gain curves is entirely contradictory with the specific wear rate curves of blank samples at the same pressures. Specifically, this is due to the high level of heat generated during the process of plastic deformation. Moreover, oxidation created a solid oxide friction film that reduced the wear rate at high friction temperature gain, and vice versa. The recorded friction temperature gains of PW samples are plotted in Figure 12b. As it can be seen from the graph below, the friction temperature gains of PW samples rise as the pressure increases from 50 to 200 MPa, and slightly declines at a pressure 350 MPa. The friction temperature gains that were recorded by the SA and the PW+SA samples, respectively, can be seen in Figure 12c,d. As pressure rises from 50 to 350 MPa, the friction temperature increases, as shown in Figure 12c. There is also a converging pattern to the changes in friction temperature gains between SA samples at different pressures. According to Figure 12d, the friction temperature gains of PW+SA samples decrease with increasing pressure from 50 to 200 MPa, and increase at a pressure 350 MPa until it reaches the same gain at 50 MPa (i.e., the slope of the friction temperature gain curves of PW+SA samples at pressure 50 MPa are more identical that ones at pressure of 350 MPa). A review of the previous data suggests that the variance in the friction temperature gain curves is more consistent with the specific wear rate curves of the PW, SA, and PW+SA samples at the same pressures.

## 4. Conclusions

Different solid lubricants (i.e., PW, SA, and their composites) were adopted to improve the compressibility and final properties of Cu–15Fe alloys that were fabricated using a PM route. Different compression pressures were also examined. The densification behavior, hardness, and tribological properties were studied. Using SEM and XRD, morphological changes were analyzed. The results showed that PW is an effective lubricant at low compaction pressures because it improves the densification behavior and shrinks pores uniformly. Specifically, the PW samples at 50 MPa have a 5.7% higher sintered density and a 38.6% higher hardness compared to blank samples. Additionally, a lower area porosity of 0.5% was observed in the PW samples. On the other hand, the hardness and sintered density properties were found to degrade when PW lubricant was used at high compaction pressures. Conversely, under a high compaction pressure of 350 MPa, the properties of the final product were improved by adding SA as a lubricant, in terms of sintering density (increasing by 2%), area porosity percentage (0.6%), the distribution of pores, and hardness (improvement by 13.5%). The SEM micrographs and corresponding EDS analysis showed the uniformly homogenous distribution of the iron-rich phase in the copper matrix in all samples. The blank sample exhibited a very low and largely unstable coefficient of friction coupled with a high specific wear rate at low pressure, and the coefficient of friction began to increase with increasing pressure. However, this behavior remained nearly unchanged in the PW sample, as the coefficient of friction was very low at low pressure but increased with increasing pressure. For instance, the PW sample has the highest specific wear rate at a pressure of 200 MPa. At 50 and 200 MPa, SA samples have a higher friction coefficient than at 350 MPa. Additionally, SA samples exhibit a nearly 50% reduction in wear rate compared to blank and PW samples. Furthermore, A 50-megapascal-pressure SA sample showed the lowest specific wear rate. The PW+SA samples have the highest values of coefficients of friction at 50 MPa, which slightly decreased with increasing pressure. In all cases, the specific wear rates of PW+SA samples were lower than those of the blank and PW samples, and were higher than those of the SA samples.

The research presented in this article can be extended in the future to include studying the effect of various types and amounts of lubricant, as well as investigating the effects of different sintering temperatures and times on final product properties.

## Figures and Tables

**Table 1:** The mixture ratio of composite lubricant and the amount of composite lubricant in Cu–15Fe based powder.

Sample	Types and Percentage of Lubricant (wt.%)		Amount of Lubricant in Cu–15Fe Composite Powder (wt.%)
	Paraffin Wax (PW)	Stearic Acid (SA)	
Blank	0	0	2
PW	100	0	2
SA	0	100	2
PW+SA	50	50	2

**Table 2:** Percentage of area porosity of sintered Cu–15Fe powder compacts with varying lubricant types under different pressures.

Samples	50 MPa (%)	200 MPa (%)	350 MPa (%)
Blank	0.86	1.24	1.1
PW	0.5	0.77	0.85
SA	0.9	0.8	0.6
PW+SA	0.7	0.81	1.1

## Data Availability

The data produced in this study are available from the authors upon reasonable request.
